# 3,3′-[1,2-Phenyl­enebis(methyl­ene)]bis­(1-octylbenzimidazolium) dibromide monohydrate

**DOI:** 10.1107/S1600536812008331

**Published:** 2012-03-03

**Authors:** Rosenani A. Haque, Muhammad Adnan Iqbal, Hoong-Kun Fun, Suhana Arshad

**Affiliations:** a School of Chemical Sciences, Universiti Sains Malaysia, 11800-Penang, Malaysia; b X-ray Crystallography Unit, School of Physics, Universiti Sains Malaysia, 11800 USM, Penang, Malaysia

## Abstract

In the title hydrated mol­ecular salt, C_38_H_52_N_4_^2+^·2Br^−^·H_2_O, the central benzene ring of the dication makes dihedral angles of 89.47 (13) and 72.69 (12)° with the pendant benzimidazol-3-ium rings. The conformations of the octyl side chains are completely different. In the crystal, the components are linked by O—H⋯Br, C—H⋯Br and C—H⋯O hydrogen bonds into a two-dimensional network lying parallel to the *ac* plane. Aromatic π–π stacking inter­actions are also observed [shortest centroid-to-centroid separation = 3.5047 (16) Å].

## Related literature

For related structures, see: Haque *et al.* (2012 ▶); Iqbal *et al.* (2012 ▶); Haque *et al.* (2011 ▶). For the stability of the temperature controller used for the data collection, see: Cosier & Glazer (1986 ▶).
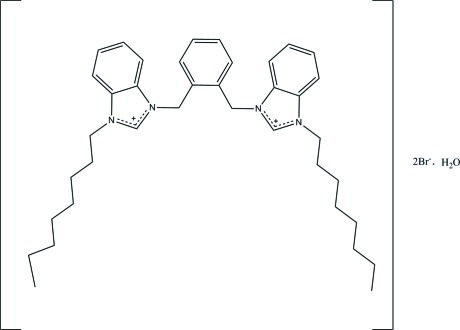


## Experimental

### 

#### Crystal data

C_38_H_52_N_4_^2+^·2Br^−^·H_2_O
*M* *_r_* = 742.67
Triclinic, 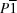

*a* = 8.7203 (4) Å
*b* = 14.9342 (12) Å
*c* = 16.4090 (8) Å
α = 115.598 (3)°
β = 104.638 (2)°
γ = 92.358 (3)°
*V* = 1836.77 (19) Å^3^
*Z* = 2
Mo *K*α radiation
μ = 2.24 mm^−1^
*T* = 100 K
0.42 × 0.32 × 0.24 mm

#### Data collection

Bruker SMART APEXII CCD diffractometer
Absorption correction: multi-scan (*SADABS*; Bruker, 2009 ▶) *T*_min_ = 0.451, *T*_max_ = 0.614
44354 measured reflections
10659 independent reflections
8816 reflections with *I* > 2σ(*I*)
*R*_int_ = 0.047

#### Refinement

*R*[*F* ^2^ > 2σ(*F* ^2^)] = 0.052
*wR*(*F* ^2^) = 0.161
*S* = 1.05
10659 reflections
416 parameters
H atoms treated by a mixture of independent and constrained refinement
Δρ_max_ = 2.62 e Å^−3^
Δρ_min_ = −1.68 e Å^−3^

### 

Data collection: *APEX2* (Bruker, 2009 ▶); cell refinement: *SAINT* (Bruker, 2009 ▶); data reduction: *SAINT*; program(s) used to solve structure: *SHELXTL* (Sheldrick, 2008 ▶); program(s) used to refine structure: *SHELXTL*; molecular graphics: *SHELXTL*; software used to prepare material for publication: *SHELXTL* and *PLATON* (Spek, 2009 ▶).

## Supplementary Material

Crystal structure: contains datablock(s) global, I. DOI: 10.1107/S1600536812008331/hb6650sup1.cif

Structure factors: contains datablock(s) I. DOI: 10.1107/S1600536812008331/hb6650Isup2.hkl

Supplementary material file. DOI: 10.1107/S1600536812008331/hb6650Isup3.cml

Additional supplementary materials:  crystallographic information; 3D view; checkCIF report

## Figures and Tables

**Table 1:** Hydrogen-bond geometry (Å, °)

*D*—H⋯*A*	*D*—H	H⋯*A*	*D*⋯*A*	*D*—H⋯*A*
O1*W*—H1*W*1⋯Br1	0.83 (5)	2.50 (5)	3.326 (3)	173 (4)
O1*W*—H2*W*1⋯Br2	0.83 (5)	2.52 (5)	3.343 (3)	177 (5)
C7—H7*A*⋯Br1	0.95	2.69	3.569 (3)	154
C15—H15*A*⋯Br1	0.99	2.92	3.672 (3)	134
C16—H16*A*⋯Br1	0.95	2.79	3.594 (3)	143
C2—H2*A*⋯Br2^i^	0.95	2.81	3.698 (3)	155
C4—H4*A*⋯O1*W*^ii^	0.95	2.49	3.218 (4)	133
C8—H8*A*⋯Br1^iii^	0.99	2.80	3.779 (3)	169
C8—H8*B*⋯Br2^iii^	0.99	2.71	3.655 (3)	159
C19—H19*A*⋯Br2^iv^	0.95	2.84	3.770 (3)	167
C21—H21*A*⋯Br2^v^	0.95	2.90	3.785 (3)	155
C31—H31*A*⋯Br2^v^	0.99	2.87	3.786 (3)	154

Symmetry codes: (i) 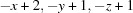; (ii) 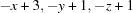; (iii) 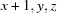; (iv) 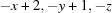; (v) 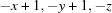.

**Fig. 1.**
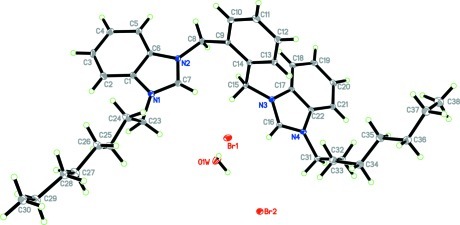
The molecular structure of the title compound, showing 30% probability displacement ellipsoids.

**Fig. 2.**
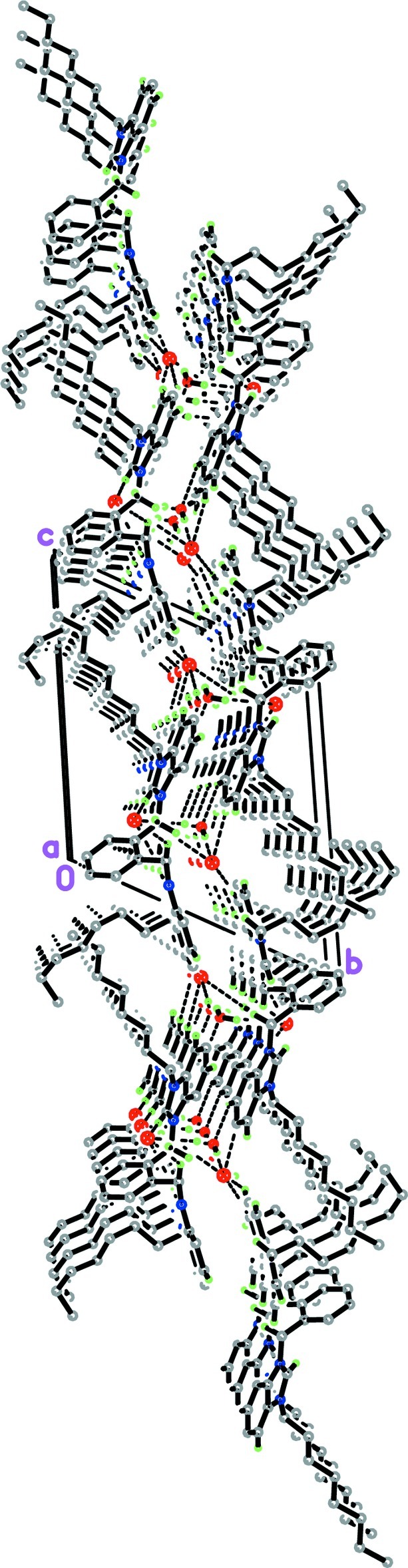
The crystal packing of the title compound. Those H atoms not involved in the intermolecular interactions (dashed lines) have been omitted for clarity.

## supplementary crystallographic information

### Comment

As a part of our ongoing studies, we have previously reported crystal structures of *ortho*-xylyl linked bis-benzimidazolium salts with ethyl (Haque *et al.*, 2012), propyl (Iqbal *et al.*, 2012), and heptyl (Haque *et al.*, 2011) substitutions. In this paper we describe single-crystal X-ray diffraction study of the title compound, (I) (Fig. 1).

Bond lengths and angles are comparable to the related structure (Haque *et al.*, 2011). The central benzene (C9–C14) ring makes dihedral angles of 89.47 (13) and 72.69 (12)° with the terminal 1*H*-benzo[*d*]imidazol-3-ium (N1/N2/C1–C7) and (N3/N4/C16–C22) rings, respectively.

The crystal structure is shown in Fig. 2. The cations, anions and water molecules are linked by intermolecular O—H···Br, C—H···Br and C—H···O hydrogen bonds (Table 1) into a two-dimensional network parallel to the *ac* plane. π–π interactions of *Cg*1···*Cg*3 = 3.5622 (18) Å (symmetry code: 3 - *x*, 1 - *y*, 1 - *z*), *Cg*3···*Cg*3 = 3.7158 (18) Å (symmetry code: 3 - *x*, 1 - *y*, 1 - *z*), *Cg*2···*Cg*4 = 3.6206 (17) Å (symmetry code: 2 - *x*, 1 - *y*, -*z*) and *Cg*4···*Cg*4 = 3.5047 (16) Å (symmetry code: 2 - *x*, 1 - *y*, -*z*) further stabilized the structure. [*Cg*1, *Cg*2, *Cg*3 and *Cg*4 is the centroid of the N1/N2/C1/C6/C7, N3/N4/C16/C17/C22, C1–C6 and C17–C22 rings, respectively].

### Experimental

A mixture of benzimidazole (5.90 g, 50 mmol) and finely ground potassium hydroxide (4.50 g, 80 mmol) in 50 ml of DMSO was stirred at room temperature (27–28 °C) for 30 min. 1-Bromoctane (8.70 ml, 50 mmol) was added drop-wise into this consistently stirred mixture with further stirring for 2 h at the same temperature. The mixture was then poured into water (700 ml) and was extracted by chloroform (5 × 30 ml). The extract was dried by filtering through five plies of Whatman filter papers. This process was repeated twice to collect crystal a clear solution which was evaporated under reduced pressure to get *N*-octylbenzimidazole (1) as a thick yellowish fluid. Furthermore, a mixture of 1 (4.04 g, 20 mmol) and 1,2-bis(bromomethyl)benzene (2.64 g, 10 mmol) in 1,4-dioxane (50 ml) was refluxed at 100 °C for 18 h. After cooling the reaction mixture to room temperature, the desired compound (2.2Br) appeared as white crystalline powder. The salt was filtered and washed by fresh 1,4-dioxane (3 × 5 ml), dried at room temperature for 24 h. The product was collected as white crystalline powder (7.42 g, 97.76%). Saturated solution of 2.2Br in methanol (0.5 ml) was exposed to diethyl ether vapours (vapour diffusion) at room temperature to get colourless blocks of (I). Single crystals were also obtained by slow evaporation of saturated solution of 2.2Br in MeOH/CH_3_CN (70:30) and by evaporating saturated solution of title compound in *d_6_*-DMSO at room temperature.

### Refinement

The H atoms of the water molecule were located in a difference Fourier map and refined freely [O—H = 0.84 (5) and 0.83 (5) Å]. All the other H atoms were positioned geometrically [C—H = 0.95–0.99 Å] and refined using a riding model with *U*_iso_(H) = 1.2 or 1.5*U*_eq_(C). A rotating group model was applied to the methyl groups.

### Crystal data

**Table:**

C_38_H_52_N_4_^2+^·2Br^−^·H_2_O	*Z* = 2
*M**_r_* = 742.67	*F*(000) = 776
Triclinic, *P*1	*D*_x_ = 1.343 Mg m^−^^3^
Hall symbol: -P 1	Mo *K*α radiation, λ = 0.71073 Å
*a* = 8.7203 (4) Å	Cell parameters from 9861 reflections
*b* = 14.9342 (12) Å	θ = 2.5–33.3°
*c* = 16.4090 (8) Å	µ = 2.24 mm^−^^1^
α = 115.598 (3)°	*T* = 100 K
β = 104.638 (2)°	Block, colourless
γ = 92.358 (3)°	0.42 × 0.32 × 0.24 mm
*V* = 1836.77 (19) Å^3^	

### Data collection

**Table:**

Bruker SMART APEXII CCD diffractometer	10659 independent reflections
Radiation source: fine-focus sealed tube	8816 reflections with *I* > 2σ(*I*)
Graphite monochromator	*R*_int_ = 0.047
φ and ω scans	θ_max_ = 30.0°, θ_min_ = 1.5°
Absorption correction: multi-scan (*SADABS*; Bruker, 2009)	*h* = −11→12
*T*_min_ = 0.451, *T*_max_ = 0.614	*k* = −20→20
44354 measured reflections	*l* = −23→23

### Refinement

**Table:**

Refinement on *F*^2^	Primary atom site location: structure-invariant direct methods
Least-squares matrix: full	Secondary atom site location: difference Fourier map
*R*[*F*^2^ > 2σ(*F*^2^)] = 0.052	Hydrogen site location: inferred from neighbouring sites
*wR*(*F*^2^) = 0.161	H atoms treated by a mixture of independent and constrained refinement
*S* = 1.05	*w* = 1/[σ^2^(*F*_o_^2^) + (0.1139*P*)^2^ + 0.460*P*] where *P* = (*F*_o_^2^ + 2*F*_c_^2^)/3
10659 reflections	(Δ/σ)_max_ < 0.001
416 parameters	Δρ_max_ = 2.62 e Å^−^^3^
0 restraints	Δρ_min_ = −1.68 e Å^−^^3^

### Special details

**Table:**

Experimental. The crystal was placed in the cold stream of an Oxford Cryosystems Cobra open-flow nitrogen cryostat (Cosier & Glazer, 1986) operating at 100.0 (1) K.
Geometry. All e.s.d.'s (except the e.s.d. in the dihedral angle between two l.s. planes) are estimated using the full covariance matrix. The cell e.s.d.'s are taken into account individually in the estimation of e.s.d.'s in distances, angles and torsion angles; correlations between e.s.d.'s in cell parameters are only used when they are defined by crystal symmetry. An approximate (isotropic) treatment of cell e.s.d.'s is used for estimating e.s.d.'s involving l.s. planes.
Refinement. Refinement of *F*^2^ against ALL reflections. The weighted *R*-factor *wR* and goodness of fit *S* are based on *F*^2^, conventional *R*-factors *R* are based on *F*, with *F* set to zero for negative *F*^2^. The threshold expression of *F*^2^ > σ(*F*^2^) is used only for calculating *R*-factors(gt) *etc*. and is not relevant to the choice of reflections for refinement. *R*-factors based on *F*^2^ are statistically about twice as large as those based on *F*, and *R*-factors based on ALL data will be even larger.

### Fractional atomic coordinates and isotropic or equivalent isotropic displacement parameters (Å^2^)

**Table:**

	*x*	*y*	*z*	*U*_iso_*/*U*_eq_	
Br1	0.86202 (3)	0.228144 (19)	0.215445 (18)	0.01929 (9)	
Br2	0.53091 (3)	0.518157 (19)	0.194634 (17)	0.01747 (9)	
N1	1.3021 (3)	0.32869 (16)	0.42917 (15)	0.0159 (4)	
N2	1.4239 (3)	0.31561 (16)	0.32400 (14)	0.0140 (4)	
N3	1.0154 (2)	0.33691 (15)	0.05850 (14)	0.0137 (4)	
N4	0.7768 (3)	0.34636 (15)	−0.01797 (14)	0.0136 (4)	
C1	1.4628 (3)	0.37063 (18)	0.47927 (17)	0.0150 (5)	
C2	1.5453 (3)	0.4119 (2)	0.57555 (18)	0.0198 (5)	
H2A	1.4928	0.4172	0.6212	0.024*	
C3	1.7092 (4)	0.4446 (2)	0.60039 (18)	0.0215 (5)	
H3A	1.7708	0.4731	0.6652	0.026*	
C4	1.7875 (3)	0.4373 (2)	0.53320 (19)	0.0201 (5)	
H4A	1.9001	0.4607	0.5539	0.024*	
C5	1.7050 (3)	0.39691 (19)	0.43801 (18)	0.0173 (5)	
H5A	1.7574	0.3923	0.3925	0.021*	
C6	1.5401 (3)	0.36325 (18)	0.41248 (16)	0.0138 (4)	
C7	1.2832 (3)	0.29643 (19)	0.33687 (18)	0.0157 (5)	
H7A	1.1844	0.2646	0.2877	0.019*	
C8	1.4578 (3)	0.30028 (19)	0.23524 (16)	0.0157 (5)	
H8A	1.5693	0.2873	0.2398	0.019*	
H8B	1.4510	0.3631	0.2289	0.019*	
C9	1.3448 (3)	0.21446 (18)	0.14737 (16)	0.0139 (4)	
C10	1.3942 (3)	0.1203 (2)	0.11448 (18)	0.0183 (5)	
H10A	1.4934	0.1124	0.1491	0.022*	
C11	1.3001 (3)	0.0386 (2)	0.03209 (19)	0.0205 (5)	
H11A	1.3348	−0.0248	0.0105	0.025*	
C12	1.1557 (3)	0.04955 (19)	−0.01872 (18)	0.0184 (5)	
H12A	1.0908	−0.0065	−0.0750	0.022*	
C13	1.1051 (3)	0.14323 (19)	0.01275 (17)	0.0172 (5)	
H13A	1.0057	0.1503	−0.0224	0.021*	
C14	1.1991 (3)	0.22640 (18)	0.09525 (16)	0.0138 (4)	
C15	1.1448 (3)	0.32824 (19)	0.13064 (17)	0.0153 (5)	
H15A	1.1069	0.3398	0.1859	0.018*	
H15B	1.2381	0.3816	0.1523	0.018*	
C16	0.8599 (3)	0.33075 (18)	0.05340 (17)	0.0146 (4)	
H16A	0.8150	0.3172	0.0948	0.018*	
C17	1.0361 (3)	0.35837 (17)	−0.01316 (16)	0.0134 (4)	
C18	1.1739 (3)	0.37683 (19)	−0.03566 (18)	0.0169 (5)	
H18A	1.2774	0.3728	−0.0023	0.020*	
C19	1.1505 (3)	0.40147 (19)	−0.10989 (18)	0.0184 (5)	
H19A	1.2409	0.4149	−0.1278	0.022*	
C20	0.9973 (3)	0.40719 (19)	−0.15933 (18)	0.0176 (5)	
H20A	0.9874	0.4241	−0.2098	0.021*	
C21	0.8599 (3)	0.38897 (18)	−0.13704 (17)	0.0160 (5)	
H21A	0.7563	0.3930	−0.1705	0.019*	
C22	0.8836 (3)	0.36425 (17)	−0.06195 (17)	0.0134 (4)	
C23	1.1749 (3)	0.3223 (2)	0.47208 (19)	0.0192 (5)	
H23A	1.0686	0.3131	0.4270	0.023*	
H23B	1.1887	0.3865	0.5298	0.023*	
C24	1.1778 (4)	0.2361 (2)	0.4980 (2)	0.0234 (6)	
H24A	1.2903	0.2349	0.5290	0.028*	
H24B	1.1358	0.1715	0.4394	0.028*	
C25	1.0773 (4)	0.2460 (2)	0.56425 (19)	0.0210 (5)	
H25A	1.1296	0.3050	0.6264	0.025*	
H25B	0.9699	0.2586	0.5379	0.025*	
C26	1.0565 (4)	0.1530 (2)	0.57879 (19)	0.0216 (5)	
H26A	0.9883	0.0966	0.5187	0.026*	
H26B	1.1631	0.1337	0.5946	0.026*	
C27	0.9807 (4)	0.1681 (2)	0.65654 (19)	0.0222 (5)	
H27A	0.8804	0.1954	0.6448	0.027*	
H27B	1.0552	0.2187	0.7180	0.027*	
C28	0.9415 (3)	0.0714 (2)	0.66330 (18)	0.0199 (5)	
H28A	0.8535	0.0253	0.6060	0.024*	
H28B	1.0372	0.0380	0.6637	0.024*	
C29	0.8916 (4)	0.0871 (2)	0.75032 (19)	0.0229 (5)	
H29A	0.7910	0.1156	0.7480	0.028*	
H29B	0.9761	0.1364	0.8079	0.028*	
C30	0.8650 (4)	−0.0110 (2)	0.7569 (2)	0.0238 (6)	
H30A	0.8340	0.0025	0.8140	0.036*	
H30B	0.9647	−0.0391	0.7600	0.036*	
H30C	0.7793	−0.0594	0.7008	0.036*	
C31	0.6030 (3)	0.34999 (19)	−0.04349 (18)	0.0159 (5)	
H31A	0.5853	0.4046	−0.0619	0.019*	
H31B	0.5656	0.3663	0.0127	0.019*	
C32	0.5035 (3)	0.25096 (19)	−0.12457 (18)	0.0177 (5)	
H32A	0.5543	0.2282	−0.1761	0.021*	
H32B	0.3949	0.2629	−0.1498	0.021*	
C33	0.4862 (3)	0.1670 (2)	−0.09634 (19)	0.0215 (5)	
H33A	0.4456	0.1920	−0.0406	0.026*	
H33B	0.5938	0.1501	−0.0773	0.026*	
C34	0.3727 (3)	0.0710 (2)	−0.17547 (19)	0.0219 (5)	
H34A	0.3552	0.0241	−0.1494	0.026*	
H34B	0.2673	0.0886	−0.1977	0.026*	
C35	0.4358 (3)	0.0177 (2)	−0.2595 (2)	0.0230 (5)	
H35A	0.5445	0.0044	−0.2365	0.028*	
H35B	0.4461	0.0630	−0.2882	0.028*	
C36	0.3284 (3)	−0.0821 (2)	−0.3359 (2)	0.0237 (6)	
H36A	0.3069	−0.1240	−0.3057	0.028*	
H36B	0.2241	−0.0678	−0.3645	0.028*	
C37	0.4014 (4)	−0.1421 (2)	−0.4146 (2)	0.0252 (6)	
H37A	0.5020	−0.1603	−0.3866	0.030*	
H37B	0.3257	−0.2055	−0.4604	0.030*	
C38	0.4385 (4)	−0.0865 (3)	−0.4677 (2)	0.0323 (7)	
H38A	0.4655	−0.1332	−0.5238	0.048*	
H38B	0.5297	−0.0315	−0.4263	0.048*	
H38C	0.3441	−0.0587	−0.4872	0.048*	
O1W	0.8891 (3)	0.47541 (18)	0.29021 (17)	0.0315 (5)	
H1W1	0.877 (5)	0.414 (3)	0.275 (3)	0.043 (12)*	
H2W1	0.802 (6)	0.488 (3)	0.267 (3)	0.040 (11)*	

### Atomic displacement parameters (Å^2^)

**Table:**

	*U*^11^	*U*^22^	*U*^33^	*U*^12^	*U*^13^	*U*^23^
Br1	0.01356 (14)	0.02374 (15)	0.02573 (15)	0.00419 (10)	0.00812 (10)	0.01460 (11)
Br2	0.01520 (14)	0.02324 (14)	0.01864 (14)	0.00351 (10)	0.00706 (10)	0.01260 (11)
N1	0.0155 (10)	0.0182 (10)	0.0157 (9)	0.0032 (8)	0.0068 (8)	0.0082 (8)
N2	0.0130 (10)	0.0168 (9)	0.0127 (9)	0.0020 (7)	0.0034 (8)	0.0074 (8)
N3	0.0114 (9)	0.0164 (10)	0.0144 (9)	0.0023 (7)	0.0043 (8)	0.0080 (8)
N4	0.0114 (9)	0.0153 (9)	0.0155 (9)	0.0029 (7)	0.0063 (8)	0.0070 (8)
C1	0.0155 (11)	0.0147 (11)	0.0154 (11)	0.0032 (9)	0.0051 (9)	0.0070 (9)
C2	0.0247 (14)	0.0188 (12)	0.0164 (11)	0.0058 (10)	0.0081 (10)	0.0073 (10)
C3	0.0241 (14)	0.0207 (12)	0.0127 (11)	0.0042 (10)	−0.0013 (10)	0.0051 (10)
C4	0.0154 (12)	0.0200 (12)	0.0207 (12)	0.0018 (9)	0.0018 (10)	0.0077 (10)
C5	0.0134 (11)	0.0195 (12)	0.0172 (11)	0.0015 (9)	0.0027 (9)	0.0080 (10)
C6	0.0140 (11)	0.0137 (10)	0.0118 (10)	0.0022 (8)	0.0023 (9)	0.0051 (9)
C7	0.0144 (11)	0.0180 (11)	0.0164 (11)	0.0028 (9)	0.0049 (9)	0.0092 (9)
C8	0.0138 (11)	0.0224 (12)	0.0121 (10)	0.0011 (9)	0.0037 (9)	0.0093 (9)
C9	0.0112 (11)	0.0190 (11)	0.0126 (10)	0.0015 (9)	0.0045 (9)	0.0077 (9)
C10	0.0169 (12)	0.0237 (12)	0.0200 (12)	0.0073 (10)	0.0079 (10)	0.0134 (10)
C11	0.0256 (14)	0.0194 (12)	0.0203 (12)	0.0074 (10)	0.0110 (11)	0.0098 (10)
C12	0.0208 (13)	0.0163 (11)	0.0157 (11)	0.0012 (9)	0.0063 (10)	0.0050 (9)
C13	0.0156 (12)	0.0204 (12)	0.0142 (11)	0.0028 (9)	0.0028 (9)	0.0076 (10)
C14	0.0125 (11)	0.0173 (11)	0.0126 (10)	0.0038 (9)	0.0044 (9)	0.0073 (9)
C15	0.0125 (11)	0.0171 (11)	0.0136 (11)	0.0019 (9)	0.0010 (9)	0.0062 (9)
C16	0.0138 (11)	0.0158 (11)	0.0149 (10)	0.0027 (9)	0.0057 (9)	0.0068 (9)
C17	0.0137 (11)	0.0123 (10)	0.0132 (10)	0.0019 (8)	0.0056 (9)	0.0040 (8)
C18	0.0143 (12)	0.0171 (11)	0.0178 (11)	0.0031 (9)	0.0068 (9)	0.0056 (9)
C19	0.0181 (12)	0.0166 (11)	0.0202 (12)	0.0017 (9)	0.0108 (10)	0.0056 (10)
C20	0.0229 (13)	0.0164 (11)	0.0167 (11)	0.0051 (9)	0.0104 (10)	0.0078 (9)
C21	0.0190 (12)	0.0165 (11)	0.0136 (11)	0.0061 (9)	0.0055 (9)	0.0075 (9)
C22	0.0139 (11)	0.0131 (10)	0.0135 (10)	0.0032 (8)	0.0068 (9)	0.0047 (9)
C23	0.0191 (12)	0.0223 (12)	0.0219 (12)	0.0056 (10)	0.0129 (10)	0.0113 (10)
C24	0.0286 (15)	0.0213 (12)	0.0278 (14)	0.0077 (11)	0.0175 (12)	0.0127 (11)
C25	0.0256 (14)	0.0196 (12)	0.0229 (13)	0.0050 (10)	0.0144 (11)	0.0103 (10)
C26	0.0257 (14)	0.0201 (12)	0.0219 (13)	0.0040 (10)	0.0130 (11)	0.0089 (10)
C27	0.0271 (14)	0.0201 (12)	0.0210 (12)	0.0032 (10)	0.0128 (11)	0.0078 (10)
C28	0.0225 (13)	0.0207 (12)	0.0177 (12)	0.0036 (10)	0.0085 (10)	0.0086 (10)
C29	0.0261 (14)	0.0243 (13)	0.0204 (12)	0.0028 (11)	0.0119 (11)	0.0095 (11)
C30	0.0219 (14)	0.0300 (14)	0.0252 (13)	0.0052 (11)	0.0102 (11)	0.0160 (12)
C31	0.0106 (11)	0.0194 (11)	0.0178 (11)	0.0044 (9)	0.0052 (9)	0.0078 (9)
C32	0.0134 (11)	0.0213 (12)	0.0188 (12)	0.0032 (9)	0.0049 (9)	0.0095 (10)
C33	0.0212 (13)	0.0232 (13)	0.0211 (12)	0.0013 (10)	0.0056 (10)	0.0115 (11)
C34	0.0183 (13)	0.0229 (13)	0.0231 (13)	0.0003 (10)	0.0068 (10)	0.0093 (11)
C35	0.0210 (13)	0.0214 (13)	0.0246 (13)	−0.0001 (10)	0.0090 (11)	0.0080 (11)
C36	0.0204 (13)	0.0223 (13)	0.0286 (14)	0.0033 (10)	0.0074 (11)	0.0119 (11)
C37	0.0259 (15)	0.0211 (13)	0.0260 (14)	0.0049 (11)	0.0061 (12)	0.0093 (11)
C38	0.0359 (18)	0.0339 (16)	0.0298 (15)	0.0072 (14)	0.0134 (13)	0.0148 (13)
O1W	0.0215 (11)	0.0275 (12)	0.0366 (13)	−0.0002 (9)	−0.0042 (9)	0.0141 (10)

### Geometric parameters (Å, º)

**Table:**

N1—C7	1.338 (3)	C23—C24	1.519 (4)
N1—C1	1.390 (3)	C23—H23A	0.9900
N1—C23	1.476 (3)	C23—H23B	0.9900
N2—C7	1.337 (3)	C24—C25	1.525 (4)
N2—C6	1.395 (3)	C24—H24A	0.9900
N2—C8	1.482 (3)	C24—H24B	0.9900
N3—C16	1.334 (3)	C25—C26	1.518 (4)
N3—C17	1.395 (3)	C25—H25A	0.9900
N3—C15	1.469 (3)	C25—H25B	0.9900
N4—C16	1.331 (3)	C26—C27	1.518 (4)
N4—C22	1.395 (3)	C26—H26A	0.9900
N4—C31	1.477 (3)	C26—H26B	0.9900
C1—C2	1.393 (3)	C27—C28	1.527 (4)
C1—C6	1.394 (3)	C27—H27A	0.9900
C2—C3	1.387 (4)	C27—H27B	0.9900
C2—H2A	0.9500	C28—C29	1.522 (4)
C3—C4	1.406 (4)	C28—H28A	0.9900
C3—H3A	0.9500	C28—H28B	0.9900
C4—C5	1.380 (4)	C29—C30	1.528 (4)
C4—H4A	0.9500	C29—H29A	0.9900
C5—C6	1.397 (3)	C29—H29B	0.9900
C5—H5A	0.9500	C30—H30A	0.9800
C7—H7A	0.9500	C30—H30B	0.9800
C8—C9	1.508 (3)	C30—H30C	0.9800
C8—H8A	0.9900	C31—C32	1.523 (3)
C8—H8B	0.9900	C31—H31A	0.9900
C9—C10	1.402 (4)	C31—H31B	0.9900
C9—C14	1.407 (3)	C32—C33	1.527 (4)
C10—C11	1.386 (4)	C32—H32A	0.9900
C10—H10A	0.9500	C32—H32B	0.9900
C11—C12	1.383 (4)	C33—C34	1.532 (4)
C11—H11A	0.9500	C33—H33A	0.9900
C12—C13	1.400 (4)	C33—H33B	0.9900
C12—H12A	0.9500	C34—C35	1.519 (4)
C13—C14	1.396 (3)	C34—H34A	0.9900
C13—H13A	0.9500	C34—H34B	0.9900
C14—C15	1.520 (3)	C35—C36	1.531 (4)
C15—H15A	0.9900	C35—H35A	0.9900
C15—H15B	0.9900	C35—H35B	0.9900
C16—H16A	0.9500	C36—C37	1.529 (4)
C17—C18	1.393 (3)	C36—H36A	0.9900
C17—C22	1.397 (3)	C36—H36B	0.9900
C18—C19	1.389 (4)	C37—C38	1.515 (4)
C18—H18A	0.9500	C37—H37A	0.9900
C19—C20	1.405 (4)	C37—H37B	0.9900
C19—H19A	0.9500	C38—H38A	0.9800
C20—C21	1.387 (4)	C38—H38B	0.9800
C20—H20A	0.9500	C38—H38C	0.9800
C21—C22	1.403 (3)	O1W—H1W1	0.84 (5)
C21—H21A	0.9500	O1W—H2W1	0.83 (5)
			
C7—N1—C1	108.6 (2)	C23—C24—H24A	109.2
C7—N1—C23	126.4 (2)	C25—C24—H24A	109.2
C1—N1—C23	125.0 (2)	C23—C24—H24B	109.2
C7—N2—C6	108.2 (2)	C25—C24—H24B	109.2
C7—N2—C8	128.7 (2)	H24A—C24—H24B	107.9
C6—N2—C8	122.9 (2)	C26—C25—C24	113.0 (2)
C16—N3—C17	108.5 (2)	C26—C25—H25A	109.0
C16—N3—C15	125.8 (2)	C24—C25—H25A	109.0
C17—N3—C15	125.6 (2)	C26—C25—H25B	109.0
C16—N4—C22	108.3 (2)	C24—C25—H25B	109.0
C16—N4—C31	126.3 (2)	H25A—C25—H25B	107.8
C22—N4—C31	125.4 (2)	C27—C26—C25	113.4 (2)
N1—C1—C2	131.5 (2)	C27—C26—H26A	108.9
N1—C1—C6	106.4 (2)	C25—C26—H26A	108.9
C2—C1—C6	122.1 (2)	C27—C26—H26B	108.9
C3—C2—C1	115.6 (2)	C25—C26—H26B	108.9
C3—C2—H2A	122.2	H26A—C26—H26B	107.7
C1—C2—H2A	122.2	C26—C27—C28	113.4 (2)
C2—C3—C4	122.4 (2)	C26—C27—H27A	108.9
C2—C3—H3A	118.8	C28—C27—H27A	108.9
C4—C3—H3A	118.8	C26—C27—H27B	108.9
C5—C4—C3	121.7 (3)	C28—C27—H27B	108.9
C5—C4—H4A	119.2	H27A—C27—H27B	107.7
C3—C4—H4A	119.2	C29—C28—C27	114.5 (2)
C4—C5—C6	116.2 (2)	C29—C28—H28A	108.6
C4—C5—H5A	121.9	C27—C28—H28A	108.6
C6—C5—H5A	121.9	C29—C28—H28B	108.6
C1—C6—N2	106.8 (2)	C27—C28—H28B	108.6
C1—C6—C5	122.0 (2)	H28A—C28—H28B	107.6
N2—C6—C5	131.1 (2)	C28—C29—C30	112.1 (2)
N2—C7—N1	110.0 (2)	C28—C29—H29A	109.2
N2—C7—H7A	125.0	C30—C29—H29A	109.2
N1—C7—H7A	125.0	C28—C29—H29B	109.2
N2—C8—C9	113.9 (2)	C30—C29—H29B	109.2
N2—C8—H8A	108.8	H29A—C29—H29B	107.9
C9—C8—H8A	108.8	C29—C30—H30A	109.5
N2—C8—H8B	108.8	C29—C30—H30B	109.5
C9—C8—H8B	108.8	H30A—C30—H30B	109.5
H8A—C8—H8B	107.7	C29—C30—H30C	109.5
C10—C9—C14	119.5 (2)	H30A—C30—H30C	109.5
C10—C9—C8	117.3 (2)	H30B—C30—H30C	109.5
C14—C9—C8	123.1 (2)	N4—C31—C32	112.6 (2)
C11—C10—C9	120.8 (2)	N4—C31—H31A	109.1
C11—C10—H10A	119.6	C32—C31—H31A	109.1
C9—C10—H10A	119.6	N4—C31—H31B	109.1
C12—C11—C10	119.9 (2)	C32—C31—H31B	109.1
C12—C11—H11A	120.0	H31A—C31—H31B	107.8
C10—C11—H11A	120.0	C31—C32—C33	113.9 (2)
C11—C12—C13	120.0 (2)	C31—C32—H32A	108.8
C11—C12—H12A	120.0	C33—C32—H32A	108.8
C13—C12—H12A	120.0	C31—C32—H32B	108.8
C14—C13—C12	120.7 (2)	C33—C32—H32B	108.8
C14—C13—H13A	119.6	H32A—C32—H32B	107.7
C12—C13—H13A	119.6	C32—C33—C34	113.6 (2)
C13—C14—C9	119.0 (2)	C32—C33—H33A	108.9
C13—C14—C15	121.2 (2)	C34—C33—H33A	108.9
C9—C14—C15	119.7 (2)	C32—C33—H33B	108.9
N3—C15—C14	113.37 (19)	C34—C33—H33B	108.9
N3—C15—H15A	108.9	H33A—C33—H33B	107.7
C14—C15—H15A	108.9	C35—C34—C33	113.3 (2)
N3—C15—H15B	108.9	C35—C34—H34A	108.9
C14—C15—H15B	108.9	C33—C34—H34A	108.9
H15A—C15—H15B	107.7	C35—C34—H34B	108.9
N4—C16—N3	110.3 (2)	C33—C34—H34B	108.9
N4—C16—H16A	124.9	H34A—C34—H34B	107.7
N3—C16—H16A	124.9	C34—C35—C36	113.6 (2)
C18—C17—N3	131.4 (2)	C34—C35—H35A	108.8
C18—C17—C22	122.3 (2)	C36—C35—H35A	108.8
N3—C17—C22	106.2 (2)	C34—C35—H35B	108.8
C19—C18—C17	115.9 (2)	C36—C35—H35B	108.8
C19—C18—H18A	122.0	H35A—C35—H35B	107.7
C17—C18—H18A	122.0	C37—C36—C35	113.8 (2)
C18—C19—C20	122.0 (2)	C37—C36—H36A	108.8
C18—C19—H19A	119.0	C35—C36—H36A	108.8
C20—C19—H19A	119.0	C37—C36—H36B	108.8
C21—C20—C19	122.3 (2)	C35—C36—H36B	108.8
C21—C20—H20A	118.9	H36A—C36—H36B	107.7
C19—C20—H20A	118.9	C38—C37—C36	114.1 (3)
C20—C21—C22	115.7 (2)	C38—C37—H37A	108.7
C20—C21—H21A	122.1	C36—C37—H37A	108.7
C22—C21—H21A	122.1	C38—C37—H37B	108.7
N4—C22—C17	106.7 (2)	C36—C37—H37B	108.7
N4—C22—C21	131.4 (2)	H37A—C37—H37B	107.6
C17—C22—C21	121.8 (2)	C37—C38—H38A	109.5
N1—C23—C24	112.5 (2)	C37—C38—H38B	109.5
N1—C23—H23A	109.1	H38A—C38—H38B	109.5
C24—C23—H23A	109.1	C37—C38—H38C	109.5
N1—C23—H23B	109.1	H38A—C38—H38C	109.5
C24—C23—H23B	109.1	H38B—C38—H38C	109.5
H23A—C23—H23B	107.8	H1W1—O1W—H2W1	108 (4)
C23—C24—C25	112.0 (2)		
			
C7—N1—C1—C2	−177.9 (3)	C9—C14—C15—N3	165.0 (2)
C23—N1—C1—C2	2.6 (4)	C22—N4—C16—N3	−0.3 (3)
C7—N1—C1—C6	0.7 (3)	C31—N4—C16—N3	−177.1 (2)
C23—N1—C1—C6	−178.8 (2)	C17—N3—C16—N4	0.4 (3)
N1—C1—C2—C3	178.2 (3)	C15—N3—C16—N4	177.2 (2)
C6—C1—C2—C3	−0.2 (4)	C16—N3—C17—C18	176.6 (3)
C1—C2—C3—C4	0.2 (4)	C15—N3—C17—C18	−0.3 (4)
C2—C3—C4—C5	0.1 (4)	C16—N3—C17—C22	−0.3 (3)
C3—C4—C5—C6	−0.5 (4)	C15—N3—C17—C22	−177.2 (2)
N1—C1—C6—N2	−0.9 (3)	N3—C17—C18—C19	−176.5 (2)
C2—C1—C6—N2	177.9 (2)	C22—C17—C18—C19	0.0 (4)
N1—C1—C6—C5	−178.9 (2)	C17—C18—C19—C20	−0.1 (4)
C2—C1—C6—C5	−0.1 (4)	C18—C19—C20—C21	0.2 (4)
C7—N2—C6—C1	0.8 (3)	C19—C20—C21—C22	−0.1 (4)
C8—N2—C6—C1	175.7 (2)	C16—N4—C22—C17	0.1 (3)
C7—N2—C6—C5	178.5 (3)	C31—N4—C22—C17	177.0 (2)
C8—N2—C6—C5	−6.5 (4)	C16—N4—C22—C21	−176.8 (2)
C4—C5—C6—C1	0.5 (4)	C31—N4—C22—C21	0.0 (4)
C4—C5—C6—N2	−177.0 (2)	C18—C17—C22—N4	−177.1 (2)
C6—N2—C7—N1	−0.3 (3)	N3—C17—C22—N4	0.1 (3)
C8—N2—C7—N1	−174.9 (2)	C18—C17—C22—C21	0.2 (4)
C1—N1—C7—N2	−0.3 (3)	N3—C17—C22—C21	177.4 (2)
C23—N1—C7—N2	179.2 (2)	C20—C21—C22—N4	176.5 (2)
C7—N2—C8—C9	−29.6 (4)	C20—C21—C22—C17	−0.1 (3)
C6—N2—C8—C9	156.5 (2)	C7—N1—C23—C24	101.5 (3)
N2—C8—C9—C10	−94.4 (3)	C1—N1—C23—C24	−79.1 (3)
N2—C8—C9—C14	89.2 (3)	N1—C23—C24—C25	165.2 (2)
C14—C9—C10—C11	−0.9 (4)	C23—C24—C25—C26	170.9 (2)
C8—C9—C10—C11	−177.4 (2)	C24—C25—C26—C27	170.5 (3)
C9—C10—C11—C12	0.0 (4)	C25—C26—C27—C28	173.2 (2)
C10—C11—C12—C13	0.4 (4)	C26—C27—C28—C29	170.3 (2)
C11—C12—C13—C14	0.1 (4)	C27—C28—C29—C30	−176.1 (2)
C12—C13—C14—C9	−0.9 (4)	C16—N4—C31—C32	−98.8 (3)
C12—C13—C14—C15	−179.4 (2)	C22—N4—C31—C32	84.9 (3)
C10—C9—C14—C13	1.4 (4)	N4—C31—C32—C33	73.6 (3)
C8—C9—C14—C13	177.6 (2)	C31—C32—C33—C34	174.3 (2)
C10—C9—C14—C15	179.9 (2)	C32—C33—C34—C35	66.7 (3)
C8—C9—C14—C15	−3.8 (4)	C33—C34—C35—C36	176.1 (2)
C16—N3—C15—C14	102.7 (3)	C34—C35—C36—C37	−173.0 (2)
C17—N3—C15—C14	−80.9 (3)	C35—C36—C37—C38	−59.2 (4)
C13—C14—C15—N3	−16.5 (3)		

### Hydrogen-bond geometry (Å, º)

**Table:**

*D*—H···*A*	*D*—H	H···*A*	*D*···*A*	*D*—H···*A*
O1*W*—H1*W*1···Br1	0.83 (5)	2.50 (5)	3.326 (3)	173 (4)
O1*W*—H2*W*1···Br2	0.83 (5)	2.52 (5)	3.343 (3)	177 (5)
C7—H7*A*···Br1	0.95	2.69	3.569 (3)	154
C15—H15*A*···Br1	0.99	2.92	3.672 (3)	134
C16—H16*A*···Br1	0.95	2.79	3.594 (3)	143
C2—H2*A*···Br2^i^	0.95	2.81	3.698 (3)	155
C4—H4*A*···O1*W*^ii^	0.95	2.49	3.218 (4)	133
C8—H8*A*···Br1^iii^	0.99	2.80	3.779 (3)	169
C8—H8*B*···Br2^iii^	0.99	2.71	3.655 (3)	159
C19—H19*A*···Br2^iv^	0.95	2.84	3.770 (3)	167
C21—H21*A*···Br2^v^	0.95	2.90	3.785 (3)	155
C31—H31*A*···Br2^v^	0.99	2.87	3.786 (3)	154

Symmetry codes: (i) −*x*+2, −*y*+1, −*z*+1; (ii) −*x*+3, −*y*+1, −*z*+1; (iii) *x*+1, *y*, *z*; (iv) −*x*+2, −*y*+1, −*z*; (v) −*x*+1, −*y*+1, −*z*.
